# Supplementary figures and images for: A spiking neural network model for fractional proprioceptive encoding of limb posture and movement in insects
Source: Biol Cybern. 2026 Feb 28;120(2):6. doi: 10.1007/s00422-025-01032-2 (PMC12950026; doi:10.1007/s00422-025-01032-2)

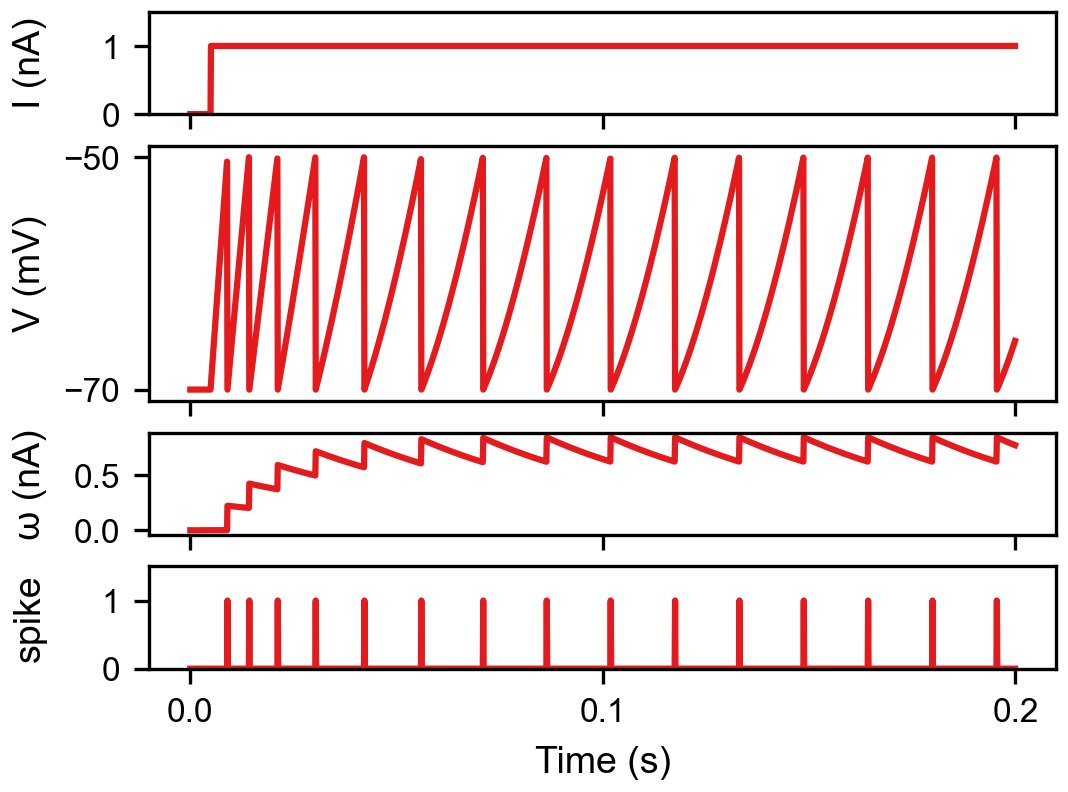

Supplement: Supplementary file 1 — Supplementary Material 1: The AdEx neuron dynamics.The AdEx model response to a sustained current, I, governed by Eqs. (6, 7, 8). This current is integrated into the membrane voltage, V. When $V = V_\text {T} = {-50} \hbox { mV}$, the membrane voltage spikes and resets to $E_\text {L} = {-70} \hbox { mV}$. During a spike event, 0.264 nA is added to $\omega$, counteracting the input current. The model exhibits lower spike frequencies with increasing $\omega$ until reaching an equilibrium spike rate. The model parameters are given in Table 1 (sensory neuron). [file 422_2025_1032_MOESM1_ESM.png]

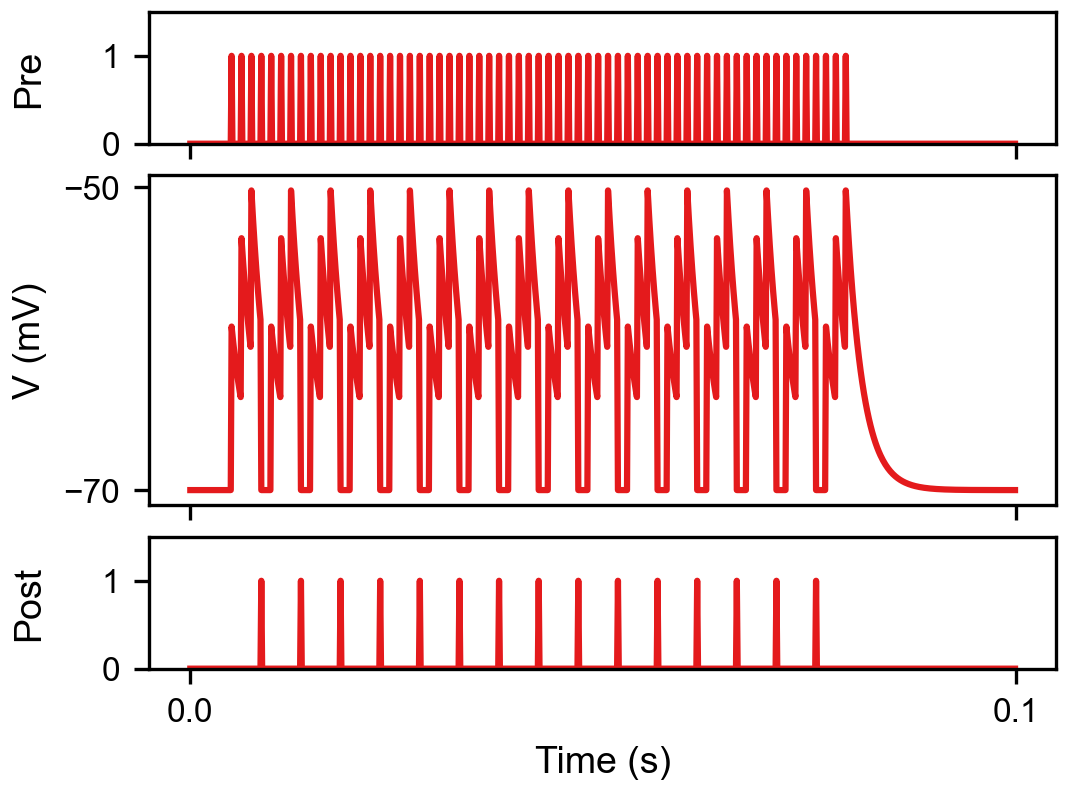

Supplement: Supplementary file 2 — Supplementary Material 2: The LIF model dynamics. The LIF model responds to a constant spike rate (333.3 Hz), governed by Eqs. (9, 10, 11). A presynaptic spike, Pre, increases the membrane potential by 10 mV, and if no spikes are present, the membrane voltage, V, decays back to $E_\text {L} = {-70} \hbox { mV}$. When $V> V_\text {T} = {-50} \hbox { mV}$, a postsynaptic spike, Post, is recorded, and V resets to $E_\text {L} = {-70} \hbox { mV}$. The postsynaptic spike rate remains constant in response to a constant presynaptic spike rate, since the LIF model has no adaptation. At the shown input spike rate, every third presynaptic spike triggers a postsynaptic spike. The model parameters are given in Table 1 (velocity neuron) [file 422_2025_1032_MOESM2_ESM.png]

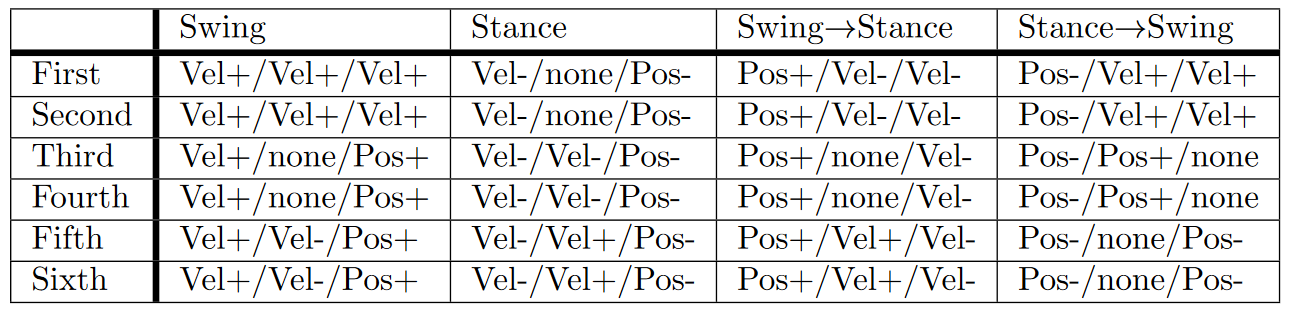

Supplement: Supplementary file 3 — Supplementary Material 3: The AdEx model response to varying a. The AdEx model’s spike response to a ramp-and-hold hair deflection at an angular velocity of $\frac{\circ }{\textrm{s}}$. The parameters for the AdEx model were taken from Table 1. The parameter a was varied to demonstrate that the tonic steady-state frequency can be reduced while maintaining a strong phasic peak. A purely phasic response can be achieved by combining the AdEx model with a high-pass filter in series. [file 422_2025_1032_MOESM3_ESM.png]

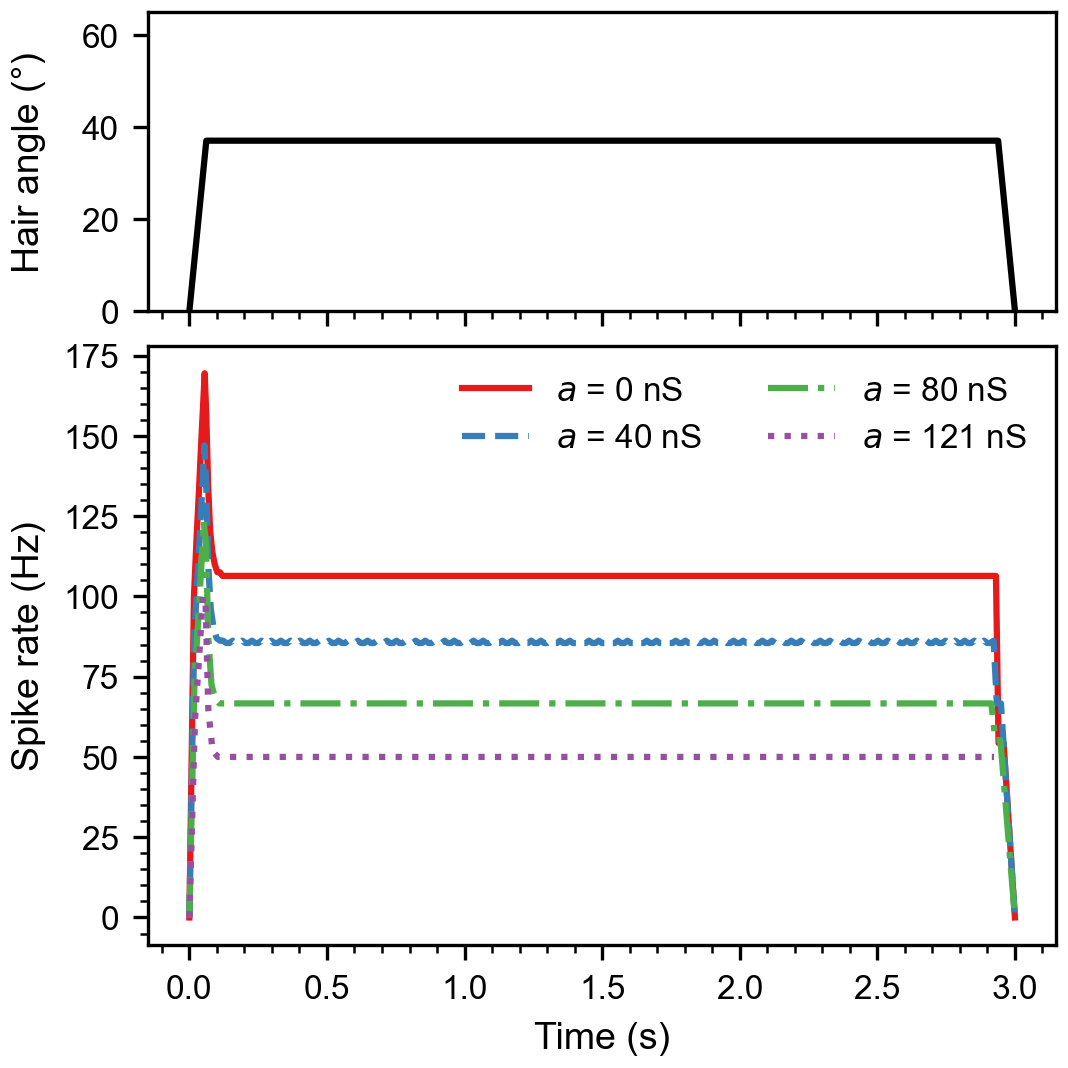

Supplement: Supplementary file 4 — Supplementary Material 4: The AdEx model response to varying C and a. The AdEx model’s spike response to a ramp-and-hold hair deflection at an angular velocity of $\frac{\circ }{\textrm{s}}$. The parameters for the AdEx model were taken from Table 1. The parameters C and a were varied to demonstrate that the strength of the phasic peak can be adjusted without altering $f_\text {ss}$ [file 422_2025_1032_MOESM4_ESM.png]

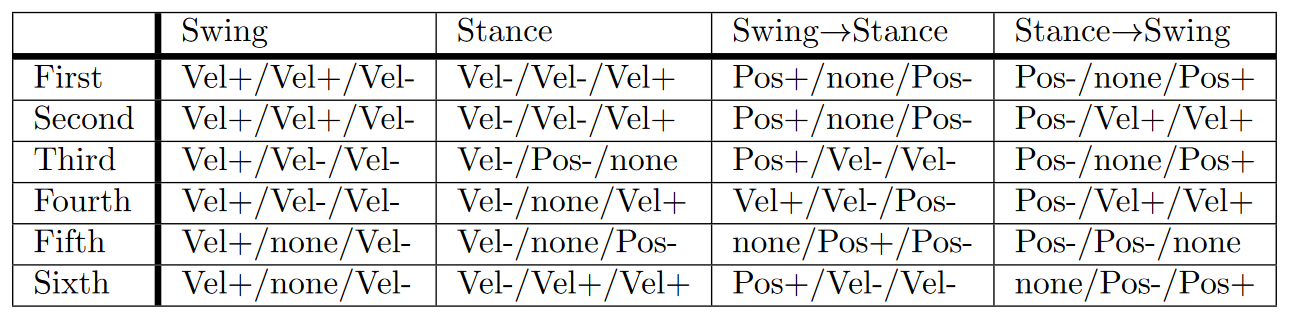

Supplement: Supplementary file 5 — Supplementary Material 5: The AdEx model response to varying C. The AdEx model’s spike response to a ramp-and-hold hair deflection at an angular velocity of $\frac{\circ }{\textrm{s}}$. The parameters for the AdEx model were taken from Table 1, with b set to zero to eliminate adaptation and yield a purely tonic response. The capacitance C was adjusted to achieve different values of $f_\text {ss}$. [file 422_2025_1032_MOESM5_ESM.png]

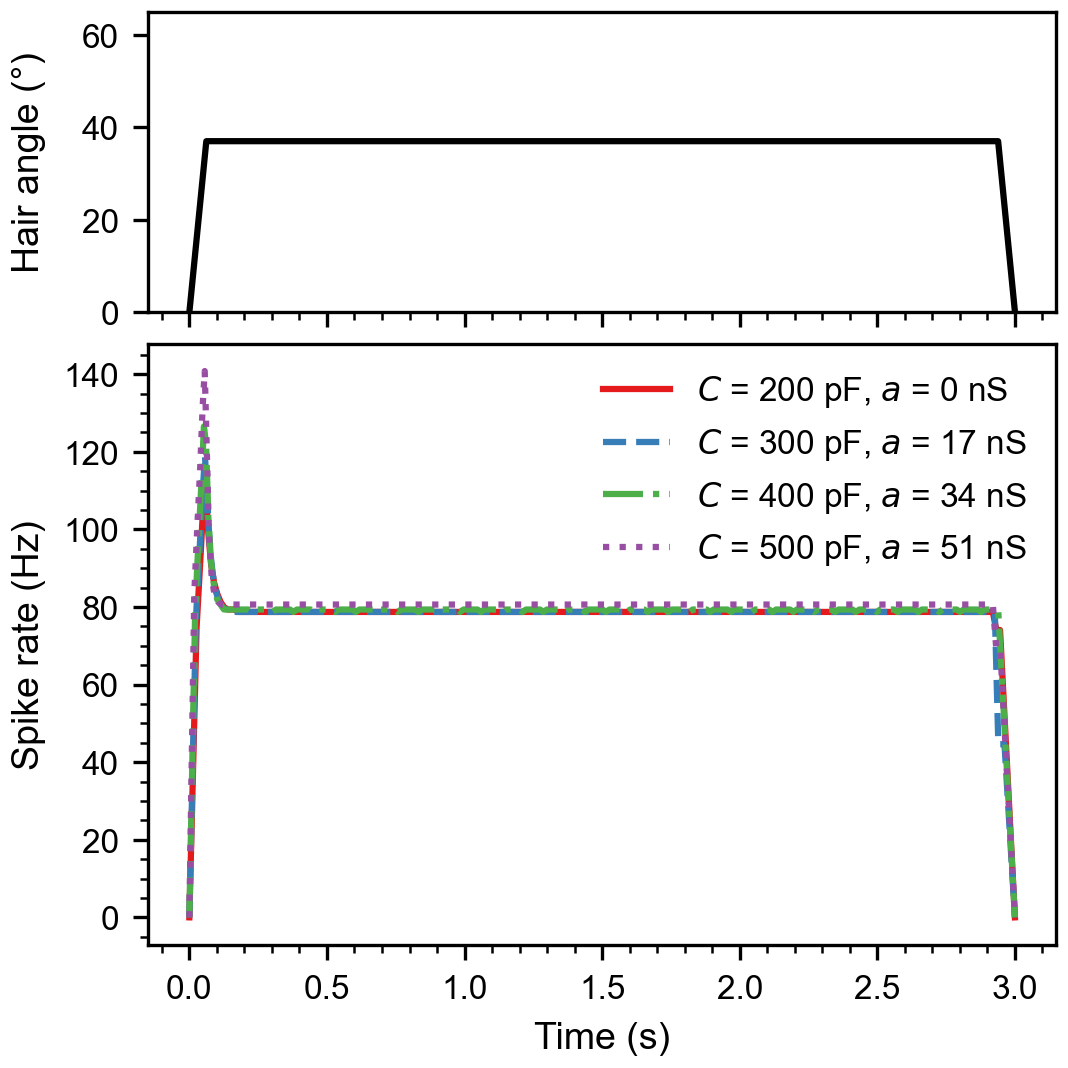

Supplement: Supplementary file 6 — Supplementary Material 6 [file 422_2025_1032_MOESM6_ESM.png]

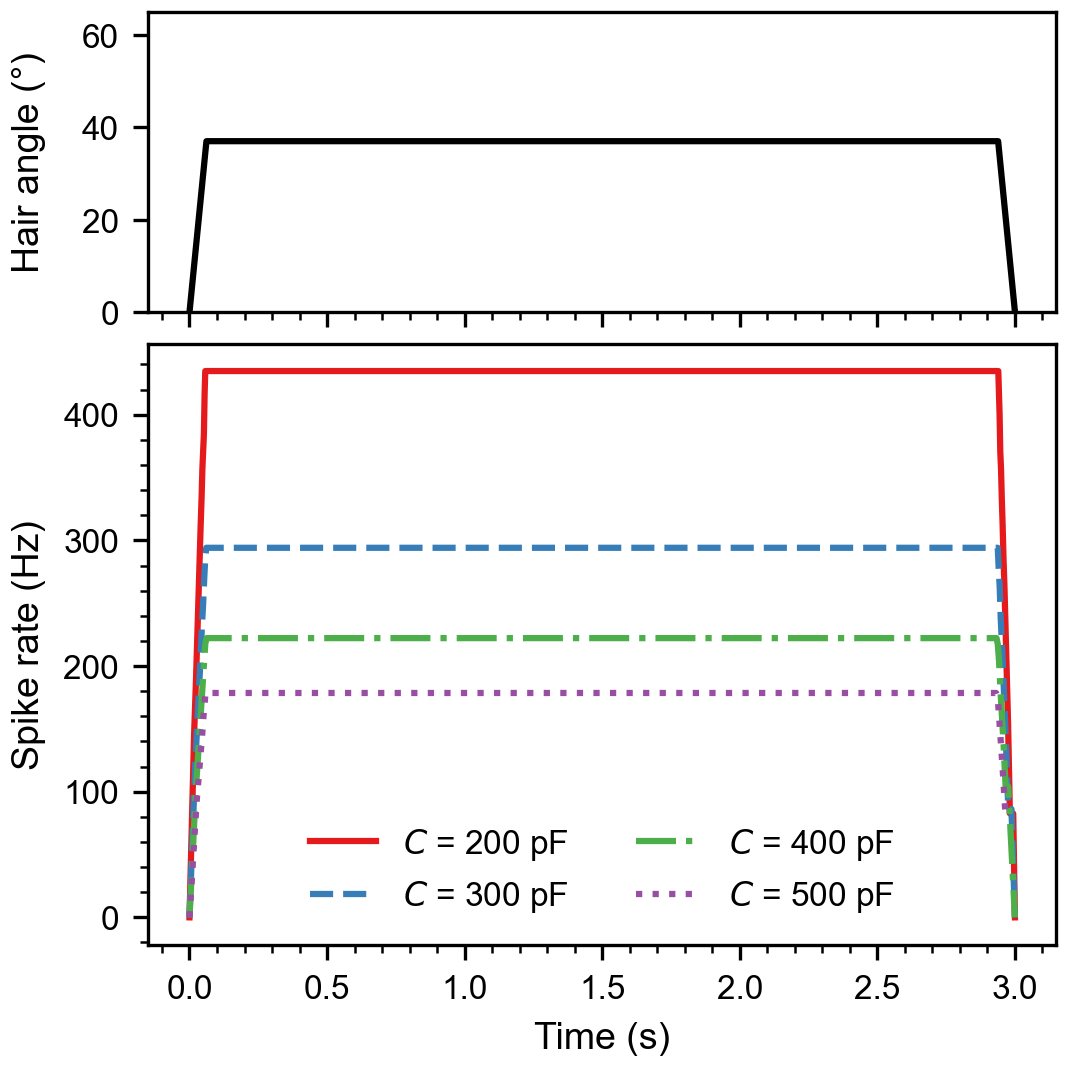

Supplement: Supplementary file 7 — Supplementary Material 7 [file 422_2025_1032_MOESM7_ESM.png]
